# Moderately hypofractionated radiotherapy for localized prostate cancer: updated long-term outcome and toxicity analysis

**DOI:** 10.1007/s00066-020-01678-w

**Published:** 2020-08-24

**Authors:** Jörg Tamihardja, Max Schortmann, Ingulf Lawrenz, Stefan Weick, Klaus Bratengeier, Michael Flentje, Matthias Guckenberger, Bülent Polat

**Affiliations:** 1 Department of Radiation Oncology, University Hospital Wuerzburg, University of Wuerzburg, Wuerzburg, Germany; 2 Department of Radiation Oncology, University Hospital Zurich, University of Zurich, Zurich, Switzerland

**Keywords:** Simultaneous integrated boost, Cone beam CT, Hypofractionation, Intensity-modulated radiation therapy, Image-guided radiation therapy

## Abstract

**Purpose:**

Evaluation of long-term outcome and toxicity of moderately hypofractionated radiotherapy using intensity-modulated radiotherapy (IMRT) with simultaneous integrated boost treatment planning and cone beam CT-based image guidance for localized prostate cancer.

**Methods:**

Between 2005 and 2015, 346 consecutive patients with localized prostate cancer received primary radiotherapy using cone beam CT-based image-guided intensity-modulated radiotherapy (IG-IMRT) and volumetric modulated arc therapy (IG-VMAT) with a simultaneous integrated boost (SIB). Total doses of 73.9 Gy (*n* = 44) and 76.2 Gy (*n* = 302) to the high-dose PTV were delivered in 32 and 33 fractions, respectively. The low-dose PTV received a dose (D95) of 60.06 Gy in single doses of 1.82 Gy. The pelvic lymph nodes were treated in 91 high-risk patients to 45.5 Gy (D95).

**Results:**

Median follow-up was 61.8 months. The 5‑year biochemical relapse-free survival (bRFS) was 85.4% for all patients and 93.3, 87.4, and 79.4% for low-, intermediate-, and high-risk disease, respectively. The 5‑year prostate cancer-specific survival (PSS) was 94.8% for all patients and 98.7, 98.9, 89.3% for low-, intermediate-, and high-risk disease, respectively. The 5‑year and 10-year overall survival rates were 83.8 and 66.3% and the 5‑year and 10-year freedom from distant metastasis rates were 92.2 and 88.0%, respectively. Cumulative 5‑year late GU toxicity and late GI toxicity grade ≥2 was observed in 26.3 and 12.1% of the patients, respectively. Cumulative 5‑year late grade 3 GU/GI toxicity occurred in 4.0/1.2%.

**Conclusion:**

Moderately hypofractionated radiotherapy using SIB treatment planning and cone beam CT image guidance resulted in high biochemical control and survival with low rates of late toxicity.

## Introduction

Primary radiotherapy as an established curative treatment option for localized prostate cancer, one of the most common cancer types [1], has undergone substantial changes in clinical practice. Lately, hypofractionated [2–7] and dose-escalated [8–10] approaches are on the advance to improve the therapeutic ratio, reduce cost, and shorten the duration of prostate radiation therapy.

When on-board cone beam CT (CBCT) became available in 2004, highly conformal image-guided intensity-modulated radiation therapy (IG-IMRT) made dose-escalated radiotherapy with reduced target volume margins a viable option. As one of the first centers worldwide to implement CBCT-based IG-IMRT, we postulated that by adopting a combination of CBCT, IG-IMRT with simultaneous integrated boost (SIB), tight margins, and hypofractionated dose-escalated radiotherapy, high biochemical control with reduction of gastrointestinal toxicity would be achievable. In an earlier publication, the outcome and toxicity data of the first treated patients were reported [11] but limited long-term data are available for moderately hypofractionated dose-escalated CBCT-based image-guided IMRT with simultaneous integrated boost (SIB). In this publication, matured long-term outcome and toxicity data are presented.

## Materials and methods

### Patients and treatment

This updated analysis is based on 346 consecutive patients treated between 2005 and 2015 with moderately hypofractionated intensity-modulated CBCT-based image-guided radiotherapy for localized prostate cancer. All patients had pathologically confirmed prostate cancer with risk stratification according to D’Amico et al.

As published before, radiotherapy was delivered with IG-IMRT or IG-VMAT in 33 fractions with SIB and two dose levels of 1.82 and 2.31 Gy per fraction, resulting in a prescribed PTV dose of 60.06 Gy (D95) and a PTV_Boost_ mean dose of 76.23 Gy. 32 fractions were applied only in patients with low-risk prostate cancer in 44 cases. For pelvic lymphatic radiation, the prescribed dose was 45.5 Gy (D95) with 1.82 Gy per fraction. A CTV_P−SV_ was generated consisting of the prostate and the base of the seminal vesicles, whereas CTV_P+SV_ included the prostate and the seminal vesicles. PTV_Boost_ was defined by a 5-mm margin around CTV_P−SV_ with avoidance of the rectum. The PTV was created by a 10-mm margin around CTV_P+SV_ in all but the dorsal direction, where a 7-mm margin was used. Pinnacle^3^ (Philips Radiation Oncology Systems, Fitchburg, WI, USA) was used for treatment planning. More information on target volume definition, treatment planning, and treatment delivery has been described before [11, 12].

Physician-recorded toxicity and biochemical control were assessed prospectively. Biochemical failure was defined according the Phoenix definition as nadir plus an increase of ≥2 ng/ml in prostate-specific antigen (PSA). Androgen deprivation therapy was recommended for patients with intermediate- (6 months) and high-risk disease (24–36 months) and prescribed at the discretion of the treating urologist. Assessment of toxicity during radiotherapy was performed every 2 weeks until the end of treatment, 6 weeks after treatment, and in 6‑month intervals thereafter. Two years after treatment, follow-up continued yearly. Gastrointestinal (GI) and genitourinary (GU) toxicity was scored using Common Terminology Criteria for Adverse Events (CTCAE) v4.0. Toxicity data of the earlier patient group from 2005 to 2010 was retrospectively reclassified from CTCAE v3.0 to v4.0 to achieve comparability. Acute toxicity was defined as occurring within 3 months after radiotherapy. Late toxicity assessment included the 6‑monthly and all later follow-ups.

### Statistics

Biochemical relapse-free survival, overall survival, prostate cancer-specific survival, and freedom from distant metastasis were calculated using the Kaplan–Meier method and log-rank tests were applied for analysis. For the comparison of toxicity of prostate only versus prostate and pelvic lymph node radiotherapy one-sided Fisher’s exact tests were performed. Statistical analysis was conducted using IBM SPSS v.25.0 (IBM Corp., Armonk, NY, USA). Differences were considered statistically significant in case of *p* < 0.05.

## Results

The reviewed 346 male patients had a median age of 73 (range 47–84) years and a median Karnofsky score of 90%. In reference to the risk classification of D’Amico 78, 122 and 142 patients had low-, intermediate-, and high- risk prostate cancer, respectively. Clinical characteristics are summarized in Table 1.

**Table 1:** Patient and treatment characteristics

Characteristic	All *N* = 346 (100%)	Prostate only *N* = 255 (73.7%)	Prostate + pelvic LN *N* = 91 (26.3%)
*Age, median (range) in years*	73 (47–84)	73 (47–83)	72 (52–84)
*KPS, median (range) in %*	90 (60–100)	90 (70–100)	90 (60–100)
*iPSA, median (range) in ng/ml*	8.4 (0.1–434.8)	6.7 (0.1–434.8)	24.8 (3.2–334)
*Gleason score*			
≤6	120 (34.7%)	114 (44.7%)	6 (6.6%)
7	142 (41.0%)	111 (43.5%)	31 (34.1%)
8–10	80 (23.1%)	27 (10.6%)	53 (58.2%)
N/A	4 (1.2%)	3 (1.2%)	1 (1.1%)
*T stage*			
T1	228 (65.9%)	180 (70.6%)	48 (52.7%)
T2	78 (22.5%)	57 (22.4%)	21 (23.1%)
T3	32 (9.2%)	15 (5.9%)	17 (18.7%)
T4	6 (1.7%)	2 (0.8%)	4 (4.4%)
N/A	2 (0.6%)	1 (0.4%)	1 (1.1%)
*D’Amico risk group*			
Low risk	78 (22.5%)	78 (30.6%)	0 (0.0%)
Intermediate risk	122 (35.3%)	117 (45.9%)	5 (5.5%)
High risk	142 (41.0%)	57 (22.4%)	85 (93.4%)
N/A	4 (1.2%)	3 (1.2%)	1 (1.1%)
*Androgen deprivation*			
Low risk	13/78 (16.7%)	13/78 (16.7%)	0 (0.0%)
Intermediate risk	34/122 (27.9%)	32/117 (27.4%)	2/5 (40.0%)
High risk	104/142 (73.2%)	33/57 (57.9%)	71/85 (83.5%)
N/A	4/346 (1.2%)	3/255 (1.2%)	1/91 (1.1%)

*KPS* Karnofsky score; *LN* lymph nodes; *N/A* not available; *iPSA* initial prostate-specific antigen

**Fig. 1:**
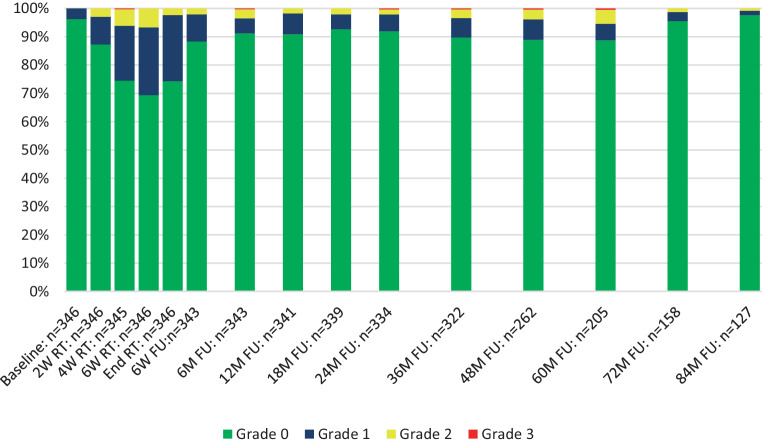
Gastrointestinal toxicity. Shown is the time course of physician-recorded gastrointestinal toxicity according to CTCAE v4.0. *RT* radiotherapy, *W* weeks, *M* months, *FU* follow-up

The time course of GI toxicity is shown in Fig. 1. No grade 4 toxicities were observed and 4 patients in total developed late grade 3 (1.2%) GI toxicity cumulated over 5‑year follow-up: 3 patients developed late rectal bleeding (mean 22.0 ± 15 months after radiotherapy, 10.0 ± 3.5 months duration) and 1 patient developed late fecal incontinence (48 months after radiotherapy, chronic); 1 patient with late rectal bleedings additionally suffered from proctitis grade 3. The maximum of acute GI toxicity occurred 6 weeks after the start of radiotherapy and decreased fast thereafter. Late grade 2 GI toxicity peaked at the 60-month follow-up and normalized thereafter. Overall, acute GI toxicity grade ≥2 was observed in 13.0% of patients and cumulative 5‑year late GI toxicity grade ≥2 in 12.1% of all patients.

**Fig. 2:**
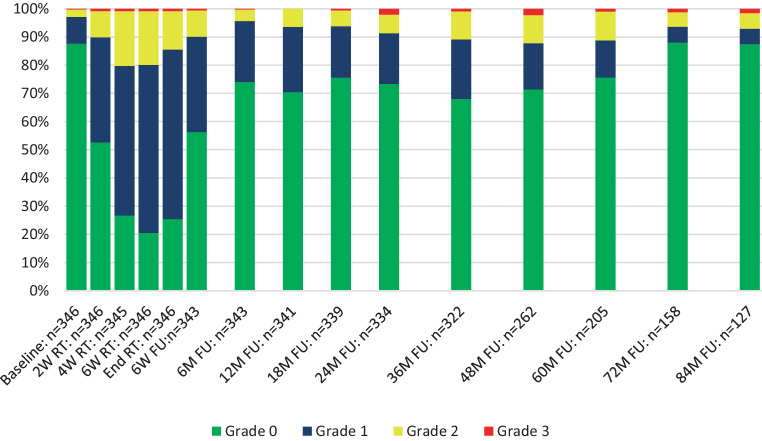
Genitourinary toxicity. Shown is the time course of physician-recorded genitourinary toxicity according to CTCAE v4.0. *RT* radiotherapy, *W* weeks, *M* months, *FU* follow-up

The time course of GU toxicity is presented in Fig. 2. No grade 4 toxicities were observed. After 5 years of follow-up, 14 patients (4.0%) developed late grade 3 GU toxicity: 5 patients developed late grade 3 macroscopic hematuria (mean 25.2 ± 14.9 months after radiotherapy, 12.0 ± 0.0 months duration; two chronic cases); 8 patients suffered from late grade 3 urinary incontinence (mean 31.5 ± 13.9 months after radiotherapy, 20.0 ± 15.0 months duration; two chronic cases); 1 patient developed late grade 3 non-infective cystitis (36 months after radiotherapy, chronic). The maximum of acute GU toxicity occurred 6 weeks after the start of radiotherapy and decreased significantly within 6 weeks. GU toxicity was increased in patients with pelvic node irradiation: cumulative 5‑year late grade ≥2 GU toxicity was observed in 23.5% of patients in the group of prostate only radiotherapy and 34.1% of the patients with prostate and pelvic lymph node irradiation (*p* = 0.036, one-sided Fisher’s exact test). Late grade 2 to 3 toxicity showed a peak at the 36-month follow-up and decreased after the 60-month follow-up. Overall acute GU toxicity grade ≥2 was observed in 30.1% and cumulative 5‑year late GU toxicity grade ≥2 in 26.3% of all patients.

**Fig. 3:**
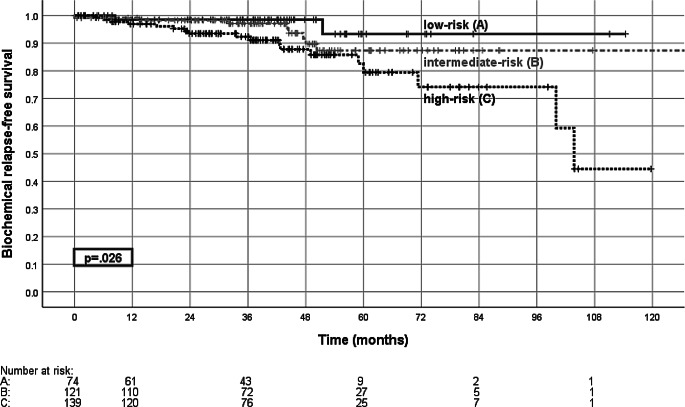
Biochemical relapse-free survival. Shown is the biochemical relapse-free survival according to risk group for the low-risk group (*A*), intermediate-risk group (*B*), and high-risk group (*C*), with the comparison of low-risk versus high-risk (*p* = 0.026, log-rank test)

**Fig. 4:**
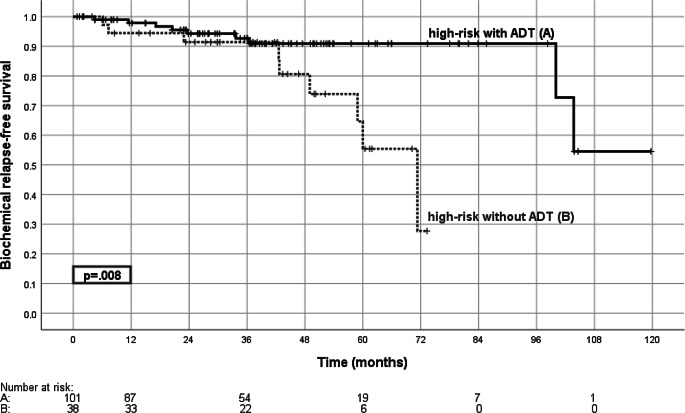
Biochemical relapse-free survival in high-risk patients with or without androgen deprivation therapy. Shown is the biochemical relapse-free survival with (*A*) and without (*B*) androgen deprivation therapy for the high-risk group (*p* = 0.008; log-rank test). *ADT* androgen deprivation therapy

Median follow-up was 61.8 (range 0.5–147.3) months. The 5‑year biochemical relapse-free survival (bRFS) was 85.4% for all patients and 93.3, 87.4, and 79.4% for low-, intermediate-, and high-risk disease (Fig. 3). For high-risk patients 5‑year bRFS was 90.9% with androgen deprivation therapy and 55.4% without (*p* = 0.008, Fig. 4). For the cohort of intermediate-risk patients androgen deprivation therapy did not significantly influence bRFS.

The 5‑year prostate-specific survival (PSS) was 94.8% for all patients and 98.7, 98.9, and 89.3% for low-, intermediate-, and high-risk disease, respectively. 10-year prostate-specific survival was 92.4%. The 5‑ and 10-year overall survival rates were 83.8 and 66.3%, respectively.

**Fig. 5:**
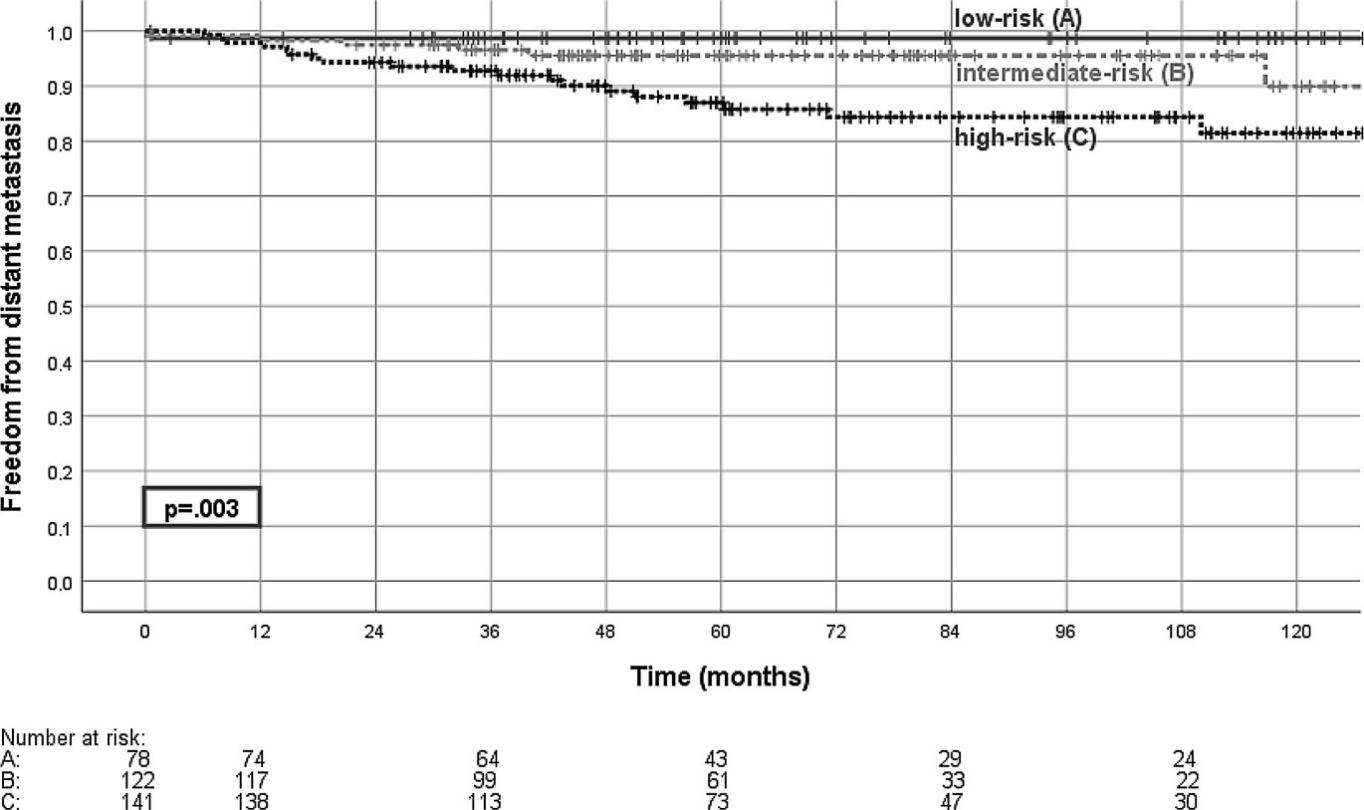
Freedom from distant metastasis. Shown is freedom from distant metastasis for the low-risk group (*A*), intermediate-risk group (*B*), and high-risk group (*C*), with the comparison of low-risk vs. high-risk group (*p* = 0.003, log-rank test)

During follow-up 27 patients developed distant metastasis, resulting in 5‑ and 10-year freedom from distant metastasis rates of 92.2 and 88%, respectively. 5‑year freedom from distant metastasis was 98.7, 95.5, and 87.0% for low-, intermediate-, and high-risk disease, respectively (Fig. 5). Androgen deprivation therapy did not significantly influence freedom from distant metastasis in any risk group.

## Discussion

Recent meta-analyses indicate that hypofractionated radiotherapy is non-inferior to conventional radiotherapy in terms of tumor control [13–15], but may suffer from an increased risk of toxicity [14, 16]. We report a “real world” cohort from a single institution. Strengths are the uniform manner of target contouring, dose prescription, and IMRT realization, as well as strict CBCT-based image guidance. The variable use of additive androgen deprivation and pelvic node irradiation in about 25% of patients with an assumed higher risk of lymph node metastasis introduces some heterogeneity which is difficult to classify.

In our patient cohort the 5‑year prostate-specific survival/biochemical relapse-free survival of 94.8/85.4% for all patients and the 5‑year bRFS of 79.4% in the high-risk group support the high efficiency of dose-escalated hypofractionated primary radiotherapy with SIB for prostate cancer. Assuming a dose–effect relationship [17, 18], this result may in part be explained by the dose prescription of 33-times 1.82/2.31 Gy. With an assumed α/β ratio of 1.5 or 2.7 Gy, this corresponds to a mean PTV_Boost_ EQD2 dose of 83 or 81 Gy, respectively. The EQD2 of 81 Gy_2.7_ in our study exceeded other recent trials of moderately hypofractionated radiotherapy like the CHHIP [2], PROFIT [19] (73 Gy_2.7_), and RTOG [4] (77 Gy_2.7_) trials, while the HYPRO [20] trial used a higher EQD2 (84 Gy_2.7_). Our outcome data are comparable to the trials mentioned above. For example, the CHHIP trial reported a 5-year bRFS of 90.6% and the PROFIT trial of 85%. While bRFS was superior in the CHHIP trial, the relative number of high-risk patients was lower than in our own patient cohort (12% vs. 41%) [2, 20]. Interestingly, for the subgroup of high-risk patients in our cohort, the 5‑year bRFS was significantly increased with androgen deprivation therapy (ADT; 5‑year bRFS 90.9% vs. 55.4%, *p* = 0.008), which clearly underlines the importance of ADT in the high-risk group [21, 22], despite treatment with escalated radiotherapy doses. In contrast, the sequence of ADT and radiotherapy appears to be less important [23]. Short-term androgen deprivation was part of the treatment protocol in the CHHIP trial; it was excluded in the PROFIT trial, which included only patients with intermediate risk. In our cohort no influence of androgen deprivation therapy on bRFS could be observed in the intermediate-risk group. Further improvements in biochemical control can be achieved by standardized androgen deprivation therapy at least in the high-risk group. Prescription of ADT in our patients was at the discretion of the treating urologist and only 73.2% of our high-risk cancer patients received ADT. Patient preference and comorbidities were considered in the decision-making process for or against ADT.

We electively irradiated the pelvic lymph nodes in patients with an estimated risk of lymph node involvement greater than 15% (according to the Roach formula) [24]. Even though evidence for prophylactic pelvic lymph node irradiation is sparse and the GETUG-01 study observed no improvement of event-free survival after a median follow-up of 11.4 years [25], the RTOG 9413 trial showed improved progression-free survival with pelvic lymph node radiation treatment [26]. Therefore, pelvic lymph node irradiation may have contributed to biochemical control in high-risk patients.

This report differs from other studies, as a simultaneous integrated boost was used and the high-dose PTV was restricted to the prostate +5-mm margin with avoidance of the rectum. The low-dose PTV (margin of 10 mm, 7 mm towards posterior) was covered by the 60 Gy isodose. To ensure precise application, an integrated offline/online protocol of volumetric cone beam CT-guided radiotherapy was strictly implemented at our institution [11, 27–30]. Accurate positioning with daily IGRT contributes to improved tumor control and minimizes dose deviations [31–33], which is especially important for small PTV margins in high-risk disease with possible microscopic extracapsular extension [34]. Our patient distribution is comparable to the Austrian-German dose-escalation trial (74 Gy for intermediate- and high-risk patients) published by Goldner et al. using 3D conformal radiotherapy [35]: while GU and GI toxicity (grade ≥2) were higher (34 and 30%), 5‑year bRFS seemed inferior (80% intermediate, 60% high risk) to our study. This underlines both the importance of technical advances (IMRT and IGRT) and of high effective doses.

Wortel et al. [36] compared the conventionally fractionated 78-Gy arms from two randomized consecutive Dutch trials using identical toxicity scoring. IG-IMRT compared to conventional 3D treatment led to a statistically significant reduction of GI toxicity ≥2 from 37.6 to 24.9%. There was at best a trend concerning GU toxicity ≥2 (36.4% vs. 46.2%). This is well in line with our results. Dose modulation using an integrated boost for the gross prostate with a tight margin may have further reduced relevant side effects.

Late GU and GI toxicity peaked between the 36-month to 60-month follow-up period consistent with earlier reports [11, 37]. We observed a late grade 3 GU toxicity rate of 4.0% and a cumulative GU toxicity ≥2 rate of 26.3% at 5 years after treatment, which seems promising considering a prescribed EQD2 > 80 Gy. Relevant structures at risk (urethra and bladder base) are always part of the anatomical CTV. Comparisons to the literature remain challenging, as particularly the reported GU toxicity rates differ considerably (grade ≥2, for example, 41.3% in the HYPRO trial [38], 29.7% RTOG 0415 trial [4], 22.2% PROFIT trial [19], 12.8% CHHIP trial [2], and 26.3% GETUG 06 trial (80 Gy) [39]). The prevalence of lower urinary tract symptoms increases in an ageing population and may influence reporting of long-term toxicity [40]. Prophylactic lymph node irradiation in 26.3% (*n* = 91) of our patients seemed to have an increased risk of GU toxicity in our series as well as in other trials [26, 41]. Overall, only 1.2% of the patients experienced late grade 3 GI toxicity and the cumulative late GI toxicity grade ≥2 rate of 12.1% remains favorable compared to randomized trials of hypofractionated radiotherapy (for example, Catton et al. 8.9% [19], Dearnarly et al. 14.7% [2], and Aluwini et al. 21.9% [38]). A low GI toxicity rate is likely attributed to our institutional policy to avoid an overlap between the organ at risk rectum and PTV_Boost_ and the definition of strict constraints for the posterior rectal wall to further limit rectal toxicity. This zero margin for the high-dose volume approach theoretically bears the risk of underdosage of dominant index lesions in the peripheral zone, but adequate dose coverage was demonstrated in an earlier publication [12].

Although dose escalation above 80 Gy might not further increase biochemical control in moderately hypofractionated radiotherapy [42], new emerging techniques aim at improving prostate cancer treatment: the ASCENDE-RT trial impressively showed a high rate of biochemical control, despite a high proportion of high-risk patients, at the cost of increased toxicity by adding LDR brachytherapy to external beam radiotherapy [43]. Another recent approach is dose escalation to the dominant index lesion as examined in the FLAME-trial. Although outcome data are pending, the published toxicity rates are promising [10]. Furthermore, recent publications of phase 3 trials [5, 6] of ultrahypofractionated regimes [44] report encouraging outcome and toxicity data with application of precise position verification systems (fiducial markers and/or catheter demarcation of the urethra) and intrafractional motion monitoring. Randomized trials are needed for the comparison of moderately hypofractionated radiotherapy with the abovementioned techniques to find the best therapeutic ratio.

## Conclusion

Moderately hypofractionated dose-escalated prostate radiotherapy using an integrated boost concept with no rectal margin for the high-dose PTV was safe and achieved high rates of long-term biochemical control and survival. Despite dose escalation, cone beam CT-based image-guided radiotherapy accomplished low rates of long-term toxicity, especially of gastrointestinal toxicity. Androgen deprivation in high-risk disease contributed to biochemical control.
